# Femtosecond laser treatment enhances DNA transfection efficiency in vivo

**DOI:** 10.1186/1423-0127-16-36

**Published:** 2009-04-01

**Authors:** Shaw-Wei D Tsen, Chao-Yi Wu, Avedis Meneshian, Sara I Pai, Chien-Fu Hung, T-C Wu

**Affiliations:** 1 Department of Pathology, The Johns Hopkins School of Medicine, Baltimore, MD 21218, USA; 2 Department of Oncology, The Johns Hopkins School of Medicine, Baltimore, MD 21218, USA; 3 Department of Molecular Microbiology and Immunology, The Johns Hopkins School of Medicine, Baltimore, MD 21218, USA; 4 Department of Obstetrics and Gynecology, The Johns Hopkins School of Medicine, Baltimore, MD 21218, USA; 5 Department of Surgery, The Johns Hopkins School of Medicine, Baltimore, MD 21218, USA; 6 Department of Otolaryngology/Head and Neck Surgery, The Johns Hopkins School of Medicine, Baltimore, MD 21218, USA

## Abstract

**Background:**

Gene therapy with plasmid DNA is emerging as a promising strategy for the treatment of many diseases. One of the major obstacles to such therapy is the poor transfection efficiency of DNA *in vivo*.

**Methods:**

In this report, we employed a very low power, near-infrared femtosecond laser technique to enhance the transfection efficiency of intradermally and intratumorally administered DNA plasmid.

**Results:**

We found that femtosecond laser treatment can significantly enhance the delivery of DNA into the skin and into established tumors in mice. In addition, we found that both laser power density as well as duration of laser treatment are critical parameters for augmenting DNA transfection efficiency. The femtosecond laser technique employs a relatively unfocused laser beam that maximizes the transfected area, minimizes damage to tissue and simplifies its implementation.

**Conclusion:**

This femtosecond new laser technology represents a safe and innovative technology for enhancing DNA gene transfer in vivo.

## Background

Gene therapy continues to evolve as an attractive approach for the treatment of many diseases (for reviews, see [1-11]). In particular, the use of plasmid DNA for gene therapy has several advantages which can circumvent the limitations and potential risks associated with viral vector-based DNA delivery. It is relatively safe, stable, and inexpensive to manufacture, making it attractive for application in the clinical arena. Furthermore, in contrast to viral vectors, DNA vaccines do not elicit anti-vector immune responses in the vaccinated patient, and, therefore, are well suited for indications likely to require multiple administrations in order to achieve and maintain target immune responses.

The ideal approach for enhancing DNA vaccine potency is by improving the transfection efficiency with minimal tissue damage. Several physical techniques including electroporation and ultrasound have been employed in an effort to improve gene transfection efficiency. However, several safety concerns have been raised with the application of these approaches in humans (for reviews, see [12,13]). Therefore, continued exploration for new methods of enhancing DNA transfection efficiency while minimizing side effects is essential for generating potent DNA vaccination strategies and also for gene therapy using plasmid DNA.

Femtosecond laser treatment represents a novel and attractive method for *in vivo *gene delivery because the lasers are convenient to operate, relatively non-invasive, and have been shown to significantly enhance gene transfection efficiency without detectable tissue damage in mice [14,15]. They have been applied toward *in vitro *genetic modification of cells (for a review, see [16]) and have recently been found to improve intradermal and intramuscular delivery of DNA in mice [14,15]. Therefore, in the current study, we employed femtosecond laser treatment in an effort to improve the transfection efficiency of DNA encoding luciferase that was administered intradermally as well as intratumorally in mice, with the hope of finding an innovative technology that may be used both for DNA vaccination as well as for plasmid DNA gene therapy in the clinical setting.

## Methods

### Laser setup

**Figure 1:**
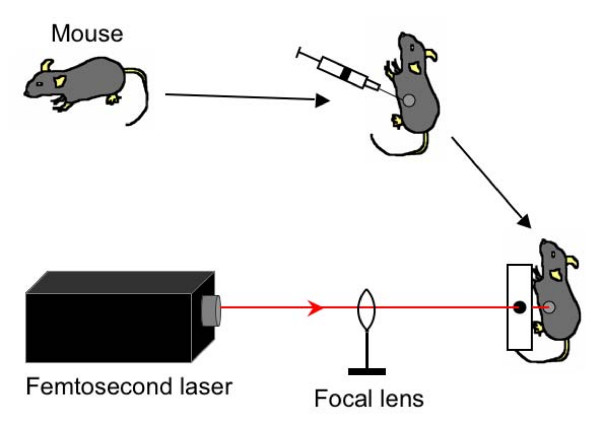
**Schematic diagram depicting the femtosecond laser setup for treatment of mice**. The laser beam was transmitted through a focal lens to allow tuning of laser power density by varying target distance from the focal lens. The beam was transmitted through a small 0.5 cm diameter hole in a wooden block to allow for optimal alignment of the laser beam with the DNA injection site on the mice.

An infrared femtosecond fiber laser system (IMRA μJewel D-400) that provides 500 fs duration pulses at ~1043 nm wavelength with repetition rate 200 kHz was used to irradiate mice (IMRA America, Inc.; Ann Arbor, MI, USA). The laser beam was transmitted through a focal lens to allow tuning of laser power density by varying target distance from the focal lens, and then through a small (0.5 cm diameter) hole in a wooden block to allow optimal alignment of the laser beam with the DNA injection site on the mice (see Figure 1).

### Plasmids and Cell Lines

The construct encoding firefly luciferase, termed pcDNA3-Luc, was a kind gift from Dr. Hyam Levitsky, Johns Hopkins University. TC-1, an E6/E7-expressing tumor cell line, was derived from primary epithelial cells of C57BL/6 mice and co-transformed with HPV-16 E6 and E7 and c-Ha-*ras *oncogenes as previously described [17].

### Intradermal gene transfection experiments

C57BL/6 mice (3 per group) were anesthetized by isofluorane inhalation, shaved, and injected intradermally (depth = 0.5 mm) with 10 μg/mouse of pcDNA3-Luc in a total volume of 50 μl. This was followed immediately by either femtosecond laser treatment at a laser power density of 0.04 GW/cm^2 ^for 80 sec or no treatment.

Nude mice were similarly injected intradermally with 10 μg/mouse of pcDNA3-Luc in a total volume of 50 μl, followed immediately by either femtosecond laser treatment at various laser power densities for 80 sec. In addition, a group of nude mice received treatment at a laser power density of 0.04 GW/cm^2 ^for different laser treatment time durations. Nude mice without laser treatment were used as control. The intensity of the luminescence in the mice was monitored by bioluminescent imaging 16 hours after DNA administration.

### Intratumoral gene transfection experiments

Female C57BL/6 mice (7 per group) were subcutaneously challenged in the abdominal wall with 1 × 10^5 ^TC-1 tumor cells/mouse. When tumors reached a diameter of ~0.7 cm, the mice were anesthetized by isoflurane inhalation, shaved, and injected intratumorally (depth = 1.0 mm) with a single dose of 10 μg/mouse of pcDNA3-Luc in a total volume of 50 μl, which was followed immediately by either femtosecond laser treatment at a laser power density of 0.04 GW/cm^2 ^for 110 sec or no treatment. The intensity of the luminescence in the mice was monitored by bioluminescent imaging 16 hours after DNA injection.

### In vivo bioluminescence imaging

To monitor luminescent intensity, the mice were injected with 0.2 ml of 15 mg/ml beetle luciferin (potassium salt, Promega) per mouse. After 10 minutes, the mice were imaged using the IVIS 200 system (Xenogen Corp, Alameda, CA). An integration time of 1 minute was used for luminescence image acquisition.

### Statistical analysis

All data expressed as means ± SE are representative of at least two different experiments. Comparisons between individual data points were made using the Student's t test. A p value < 0.05 was considered statistically significant.

## Results

### Femtosecond lasers enhance the transfection efficiency of luciferase-encoding plasmid DNA administered intradermally

**Figure 2:**
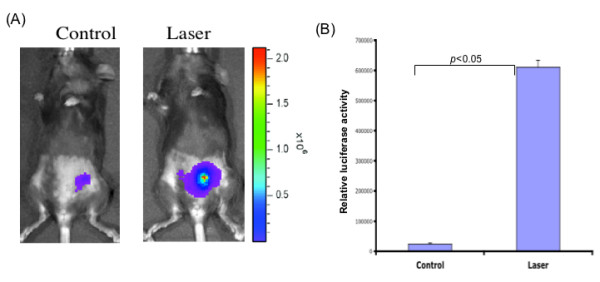
**Characterization of luciferase expression in mice injected intradermally with luciferase expressing DNA with or without laser treatment**. C57BL/6 mice (3 per group) were injected intradermally with 10 μg/mouse pcDNA3-luc in 50 μl volume. Immediately after DNA injection, the injected mice were treated with laser for 80 sec at a laser power density of 0.04 GW/cm^2^. Mice were imaged 16 hours after DNA injection using the IVIS Imaging System Series 200. Bioluminescence signals were acquired for 1 minute. **(A) **Representative luminescence images of injected mice with or without laser treatment. **(B) **Bar graph showing relative luciferase activity for both groups of mice using data from all mice tested (p < 0.05).

In order to determine if femtosecond laser treatment can improve the transfection efficiency of intradermally administered luciferase-encoding DNA plasmid, female C57BL/6 mice (3 per group) were injected intradermally with the pcDNA3-luciferase plasmid, followed immediately by femtosecond laser treatment at the injection site. As shown in Figure 2, we found that mice receiving femtosecond laser treatment after DNA administration generated significantly enhanced luciferase gene expression compared to mice injected with DNA without laser treatment (p < 0.05). Thus, our data indicate that femtosecond laser treatment can enhance the transfection efficiency of intradermally injected DNA.

### Enhancement in transfection efficiency using femtosecond laser treatment depends on the laser power density and duration of laser treatment

**Figure 3:**
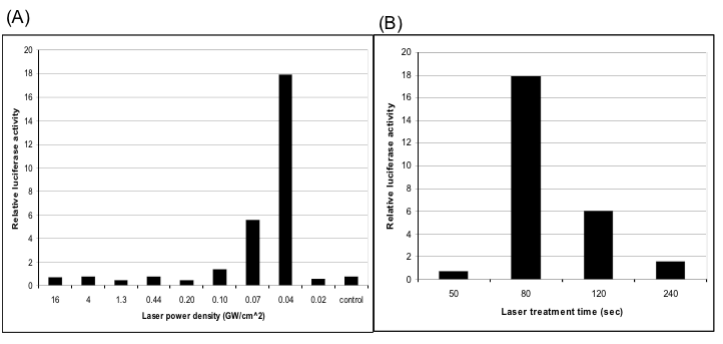
**Characterization of optimal laser power density and duration of laser treatment for maximum enhancement in transfection efficiency**. Nude mice were injected intradermally with 10 μg/mouse pcDNA3-luc in 50 μl volume and immediately treated with femtosecond laser at various laser power densities and with various laser treatment time durations. Mice were imaged 16 hours after DNA injection using the IVIS Imaging System Series 200. Bioluminescence signals were acquired for 1 minute. Laser was first optimized for laser power density, and then for laser treatment time. Untreated mice were used as control. **(A) **Bar graph showing relative luciferase activity in mice receiving laser treatment at different laser power densities, while keeping laser treatment time duration constant at 80 sec. **(B) **Bar graph showing relative luciferase activity in mice receiving laser treatment at different laser treatment time durations, while keeping laser power density at 0.04 GW/cm^2^.

In order to evaluate the maximum enhancement of transfection efficiency achievable with the application of femtosecond laser treatment with intradermally administered DNA, we modulated the laser power density and the duration of laser treatment. C57BL/6 mice (3 per group) were injected intradermally with the pcDNA3-luciferase plasmid, which was followed immediately by femtosecond laser treatment at the injection site using various laser power densities and time durations of laser treatment. As shown in Figure 3A, at a constant duration of laser treatment of 80 seconds, we found that mice receiving laser treatment at a laser power density of 0.04 GW/cm^2 ^generated the highest transfection efficiency compared to mice treated at different laser power densities. This laser power density corresponds to a laser energy of 2.6 μJ/pulse with a spot size of 4 mm. Furthermore, at a constant laser power density constant of 0.04 GW/cm^2^, we found that mice receiving laser treatment for a duration of 80 seconds generated the highest transfection efficiency compared to mice treated for different durations (Figure 3B). Treatment of DNA-administered mice with laser for 80 sec at a laser power density of 0.04 GW/cm^2 ^resulted in the highest (up to 23-fold) enhancement of gene expression compared to mice without laser treatment (see Figure 3). These results indicate that the laser power densities as well as the duration of laser treatment are critical factors affecting the enhancement of transfection efficiency of intradermally administered DNA using femtosecond laser treatment.

### Femtosecond laser treatment can enhance the transfection efficiency of luciferase-expressing DNA injected intratumorally

**Figure 4:**
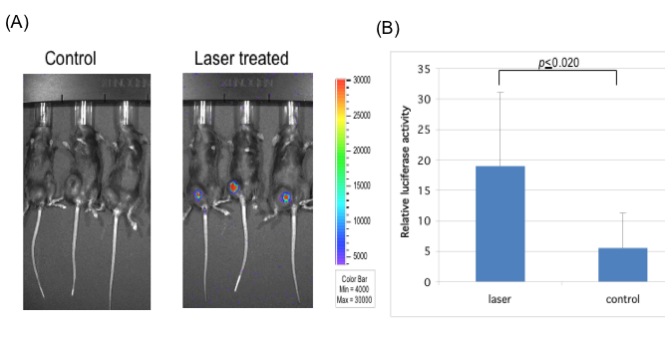
**Characterization of luciferase expression in mice injected intratumorally with luciferase expressing DNA with or without laser treatment using luminescence imaging**. C57BL/6 mice were challenged subcutaneously with 1 × 10^5^/mouse of TC-1 tumor cells/mouse in the right leg. When tumors reached a diameter of ~0.7 cm, mice were injected intratumorally with pcDNA3-Luc, followed by either femtosecond laser treatment for 110 sec at a laser power density of 0.04 GW/cm^2 ^or no treatment. Mice were injected with luciferin and imaged using the IVIS Imaging System Series 200 using methods as described previously [18]. Bioluminescence signals were acquired for 1 minute. **(A) **Representative luminescence images of injected mice with or without laser treatment. **(B) **Bar graphs depicting the quantification of luciferase activity in mice with or without laser treatment.

To determine if femtosecond laser treatment can be used to enhance the transfection efficiency of DNA administered intratumorally, we performed tumor gene transfection experiments. Female C57BL/6 mice were challenged subcutaneously with 1 × 10^5 ^of TC-1 tumor cells/mouse in the right leg. When tumors reached a diameter of ~0.7 cm, mice were injected intratumorally with pcDNA3-Luc DNA, followed by either femtosecond laser treatment for 110 seconds at a laser power density of 0.04 GW/cm^2 ^or no treatment. A laser treatment time duration of 110 seconds was chosen based on preliminary experiments (data not shown). As shown in Figure 4, we found that tumor-bearing mice injected intratumorally with DNA followed by laser treatment had significantly increased luciferase expression 16 hours after injection compared to mice injected with DNA without laser treatment (p ≤ 0.020). Thus, our data indicate that treatment with femtosecond lasers after DNA administration can enhance the transfection efficiency of DNA injected into tumors.

## Discussion

In this study, we found that femtosecond lasers can significantly enhance DNA transfection efficiency into the skin and into established tumors in mice, and that both the laser power density used as well as the laser treatment duration are important parameters that determine the enhancement in gene transfection afforded by the femtosecond laser. Using these parameters, we have optimized the laser system for maximum intradermal and intratumoral DNA transfection efficiency.

Our data are consistent with previous reports that femtosecond lasers can enhance intradermal DNA delivery [15]. However, ours is the first report demonstrating the use of femtosecond lasers to enhance intratumoral DNA delivery. Compared to these previous studies, our femtosecond laser treatment employs significantly reduced laser focusing, which offers several advantages. First, it greatly increases the transfected area and thus maximizes the number of cells that are transfected. Second, the laser energy is greatly reduced, thereby minimizing damage to healthy host tissue. Moreover, it is a relatively simple system to implement. Such characteristics make this technique especially promising for future clinical applications.

Our results suggest that femtosecond laser treatment may improve the efficacy of DNA vaccination and/or gene therapy using plasmid DNA. Such a laser technology can be used to deliver DNA encoding antitumor genes including proapoptotic, immunostimulatory, and/or antiangiogenic factors to effect eradication of tumors through apoptosis, immune-mediated mechanisms, and nutrient deprivation. In future studies, it will be important to explore the employment of laser treatment in combination with gene therapy using plasmid DNA encoding these factors for the control of disease in preclinical models.

However, the eventual clinical translation of the femtosecond laser technique would require several important considerations. Although our results demonstrated significantly improved transfection of subcutaneous tumors using laser treatment, many tumors clinically are less accessible and are located deep within body cavities. Novel adaptations must therefore be made to make these tumors accessible, such as the employment of lasers with longer wavelengths that have greater penetration depth in tissue, or percutaneous laser delivery devices which can allow laser delivery to deeper structures through minimally invasive means. In addition, the size of the laser beam can be further increased to allow the transfection of larger numbers of tumor cells, and the portability of the laser system must be improved in order to make it more clinically accessible.

## Conclusion

Our data provide a platform on which a new femtosecond laser-based DNA delivery strategy can be developed. We have determined that laser power density and duration of laser treatment play a crucial role for optimizing the transfection efficiency of DNA, and that these parameters vary depending on the location of delivery (intradermal versus intratumoral). Further optimization of these parameters will be necessary for future application of the femtosecond laser system in humans and in a broader range of tumor sites. It is our hope that such a technology will aid gene therapy with plasmid DNA and DNA vaccination for the treatment of human disease.

## Competing interests

The authors declare that they have no competing interests.

## Authors' contributions

S-WDT and C-YW were involved in the execution of the project and interpretation of the data. AM and SIP provided their expertise in the interpretation of the data. C-FH and T-CW provided overall supervision and guidance for the project. All authors read and approved the manuscript.
